# Single-cell analysis reveals chemokine-mediated differential regulation of monocyte mechanics

**DOI:** 10.1016/j.isci.2021.103555

**Published:** 2021-12-02

**Authors:** Tom M.J. Evers, Vahid Sheikhhassani, Mariëlle C. Haks, Cornelis Storm, Tom H.M. Ottenhoff, Alireza Mashaghi

**Affiliations:** 1 Medical Systems Biophysics and Bioengineering, Leiden Academic Centre for Drug Research, Faculty of Mathematics and Natural Sciences, Leiden University, Einsteinweg 55, 2333CC Leiden, the Netherlands; 2 Department of Infectious Diseases, Leiden University Medical Center, Albinusdreef 2, 2333ZC Leiden, the Netherlands; 3 Department of Applied Physics and Institute for Complex Molecular Systems, Eindhoven University of Technology, Eindhoven, the Netherlands

**Keywords:** Immunology, Cell biology, Biomechanics

## Abstract

Monocytes continuously adapt their shapes for proper circulation and elicitation of effective immune responses. Although these functions depend on the cell mechanical properties, the mechanical behavior of monocytes is still poorly understood and accurate physiologically relevant data on basic mechanical properties are lacking almost entirely. By combining several complementary single-cell force spectroscopy techniques, we report that the mechanical properties of human monocyte are strain-rate dependent, and that chemokines can induce alterations in viscoelastic behavior. In addition, our findings indicate that human monocytes are heterogeneous mechanically and this heterogeneity is regulated by chemokine CCL2. The technology presented here can be readily used to reveal mechanical complexity of the blood cell population in disease conditions, where viscoelastic properties may serve as physical biomarkers for disease progression and response to therapy.

## Highlights

• Mechanical properties of monocytes are affected by temperature changes
• Mechanical properties of monocytes are strain-rate dependent
• CCL2 affects both the viscous and elastic properties of monocytes
• CCL2 potentially modulates monocytes mechanically to transit to a primed state

## Graphical abstract

**Figure:**
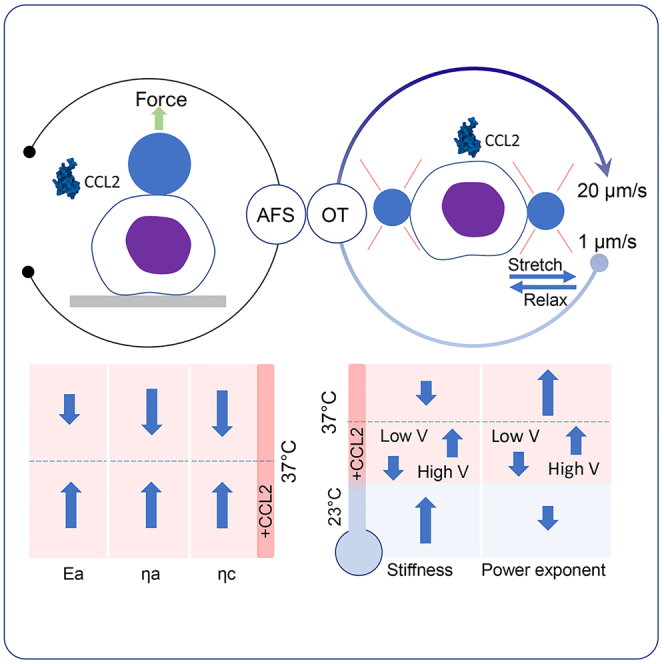

## Introduction

Accumulating evidence indicates that cell mechanical properties, such as stiffness and viscosity, are key indicators of cytoskeleton (Pegoraro et al., 2017; Walker et al., 2020) and nuclear organization (Miroshnikova et al., 2017; Stiekema et al., 2020) and may offer label-free intrinsic biophysical markers for determining physiological cell state transitions or pathological cell changes. Strikingly, little information is available on the mechanical properties of immune cells, despite the fact that these continuously adapt their mechanics in order to function properly, *e.g.* in circulation, phagocytosis, migration, and cell–cell interactions (Huse, 2017). This lack of information is mainly due to the fact that the application of mechanical probes commonly employed elsewhere in cell biophysics are relatively new to immunologists, whereas biophysicists — on the other hand — have focused mostly on the mechanics of red blood cell (RBC), among various blood cell types, owing to this model's simplicity. Application of mechanical analysis on RBCs has led to improvement in understanding disease (e.g., sickle cell (Alapan et al., 2014; Byun et al., 2012; Maciaszek and Lykotrafitis, 2011; Xu et al., 2020) and malaria (Depond et al., 2020; Hosseini and Feng, 2012; Ye et al., 2013)) and drug effects (Sheikh-Hasani et al., 2018), but has yet to be established for other blood cells among which, notably, immune cells.

Monocytes form a major subpopulation of innate immune cells that can immediately adapt to their microenvironment for pathogenesis or homeostasis by altering their mechanical properties. They are able to pass through the narrowest capillaries and bifurcations in the circulation by gradually adjusting their morphology (Dupire et al., 2020). Upon infection, monocytes need to undergo large deformations to extravasate across the endothelium, and form extensive actin-based protrusions during phagocytic engulfment of pathogens (Freeman and Grinstein, 2014). In cancer, monocytes extravasate from the vasculature into primary tumor sites, and differentiate into tumor-associated macrophages to promote tumor growth and metastasis. For both processes, chemokine CCL2 has emerged as the primary mediator of monocyte recruitment (Gschwandtner et al., 2019; Hanna et al., 2015; Qian et al., 2011). However, we lack insights into if and how CCL2 induces mechanical changes that trigger monocytes to switch from their resting state in the circulation into a migration-competent state allowing them to extravasate and enter tissue. As such, probing the mechanical properties of these cells could provide fundamentally important insights into their function in health and disease, and could provide novel biomarker of immune activation and disease.

To that end, we combined two complementary single-cell force spectroscopy approaches; the recently introduced technique of acoustic force spectroscopy (AFS) (Sitters et al., 2014) and optical tweezers (OTs), and present the first single-cell force spectroscopic characterization of monocyte mechanics. This unique platform is utilized to provide a full mechanical characterization of human monocytes, and AFS has not been applied to primary human cells before. Our approach overcomes technical barriers associated with existing techniques used for single-cell analysis, such as the low throughput of atomic force microscopy, or the high computational needs of traction force microscopy. In addition, our OT and AFS are both equipped with a temperature control system, which is of critical importance as the chemical reactions, protein functionality, and constitutive materials of the cell are inherently temperature-sensitive. The technical challenge to temperature control is a major reason why cell mechanics data at physiological temperatures are scarce in biophysics literature. Our study not only provides mechanical information of resting monocytes at physiological temperatures but also the mechanical changes in monocytes induced by one of the most important chemokines, CCL2.

## Results

### Monocyte mechanics is strain-rate dependent

**Figure 1:**
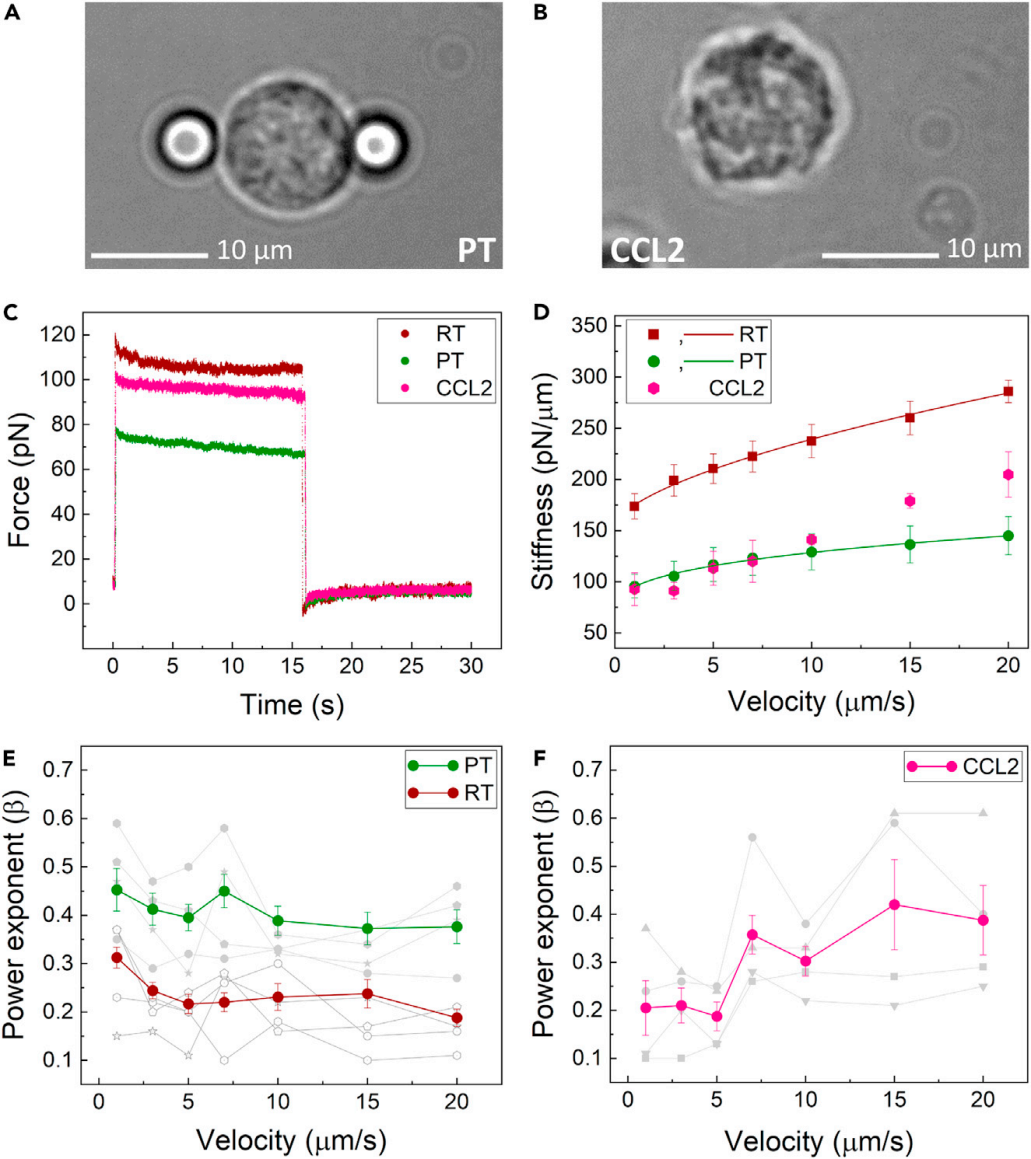
Human monocyte mechanics is affected by strain rate and CCL2 treatment (A) Schematic representation of OT, in which a monocyte is sandwiched between two optically trapped beads. (B) Morphological changes of a monocyte after treatment with 200 ng/mL of CCL2 for 15 min prior to bead attachment. (C) Typical trace of recorded force versus time at RT, PT, and upon stimulation by CCL2. (D) Dynamic stiffness of monocytes at RT, PT, and after treatment with CCL2 fitted by a power law model. CCL2-treated cells could not be fitted by the power-law function, and only data points are shown. (E) Power exponent of stress-relaxation analysis as a function of strain rate at RT and PT showed a significant difference (p < 0.0001). (F) In case of CCL2-treated cells, power exponent revealed strain-rate dependent changes; 0.20 ± 0.04 for velocities from 1 to 5 μm/s and 0.36 ± 0.03 for velocities from 7 to 20 μm/s (p value = 0.002). Data are represented as mean ± SEM.

We first set out to probe the mechanical properties of primary monocytes at room temperature (RT, 23°C) using optical tweezers (OTs). To do so, freshly isolated monocytes from healthy human blood bank donors were clamped between two optically trapped beads and subsequently stretched by displacing one of the trapped beads for 500 nm, while keeping the other bead stationary. We performed multiple stretching cycles, in which the cells were pulled at a constant strain rate at different values of velocity v=dγ/dt of 1, 3, 5, 7, 10, 15, and 20 μm/s. Upon stretching, monocytes showed a strain-rate dependent increase in stiffness (Figure 1D). At zero strain, we found an average stiffness of 154.61 ± 6.97 pN/μm. Of note, this stiffness is more than 40-fold higher compared with the stiffness we have previously measured for RBCs (Sheikh-Hasani et al., 2018). In addition to stiffness, we determined the viscoelastic behavior of monocytes by fitting their relaxation response to a power-law decay function (Figure 1E). In the power law function, a purely elastic solid exhibits a power-law exponent of 0, and a purely viscous fluid a power-law exponent of 1 (Kollmannsberger and Fabry, 2011). For monocytes at RT, we found a power law factor of 0.23 ± 0.04, indicating that compared with RBCs, monocytes at RT behave more toward the solid-like end of the viscoelastic spectrum. Next, we assessed whether the obtained RT features are physiologically relevant. Therefore, we also probed the mechanical properties of monocytes at physiological temperatures (PT, 37°C). Importantly, monocytes at PT showed significantly (p < 0.0001) reduced stiffness (defined as the linear slope of the stress-strain curve as measured at constant strain rate) at all applied strain rates, as compared with RT (Figure 1D). In addition, we observed a moderate increase in stiffness with increasing strain rates at PT, indicating a higher resistance to the applied strain for faster deformations. We also examined the degree of viscoelasticity exhibited by human monocytes, quantified by the fitted power-law exponent β. Our data show that monocytes exhibit increasingly fluid-like behavior at PT, as compared with RT (Figure 1E), indicating that temperature significantly impacts the mechanical properties of human monocytes. This finding further underlines the need for mechanical studies conducted at physiological temperature.

### CCL2 induces strain-rate dependent changes in monocyte mechanics

A common approach used to manipulate the viscoelastic properties of cells in mechanical studies is the exposure of cells to synthetic chemicals or drugs that act on the cytoskeleton, which cause disruption of actin and tubulin filaments (Fritzsche et al., 2017; Nijenhuis et al., 2014; Romanov et al., 2021; Wang et al., 2001; Weber et al., 2019). Here, we opted for an immuno-physiologically relevant approach, by exposing monocytes to one of the most important chemokines, CCL2. CCL2 is renowned for its ability to drive the chemotaxis of myeloid and lymphoid cells on short time scales during physiological immune defense. While CCL2-mediated monocyte recruitment in the context of inflammation has been extensively studied, it is not known whether and, if so, how CCL2 affects the mechanical properties of monocytes. Because monocytes are incited to migrate upon stimulation by CCL2, we hypothesized that, in response to CCL2, monocytes were likely to show altered mechanical properties that might facilitate swift coordination of cell motility.

To test this hypothesis, we treated freshly isolated human blood monocytes with either 200 ng/mLCCL2 or an equal volume of PBS, the CCL2 diluent, for 15 min at 37°C. This concentration has been documented to induce cellular chemotaxis of monocytes (Carvallo et al., 2015). We first investigated the effect of CCL2 on the mechanical properties of monocytes under different strain rates, using our OT setup. At low strain velocities (1–10 μm/s), CCL2-treated monocytes showed comparable stiffness to non-treated cells at PT (Figure 1D), whereas at higher velocities (15 and 20 μm/s) they displayed a significant (p = 0.002) increase in stiffness, suggesting that CCL2 elicits differential stiffening at elevated strain rates. Notably, the stiffness at different strain rates could not be fitted by the power-law function, signaling drastic and fundamental alterations in mechanical behavior.

To further explore these alterations, we probed the viscoelastic behavior of monocytes in response to CCL2. Strikingly, at low strain rates (1–5 μm/s) we found power factors at the same range for cells at RT, whereas at higher strain rates (7–20 μm/s) the power factor increased to values comparable with cells at PT (Figure 1F). Again, these results point to a differential sensitization to strain rate, induced by CCL2, particularly at the higher strain rates.

### CCL2 affects elastic and viscous moduli of monocytes

**Figure 2:**
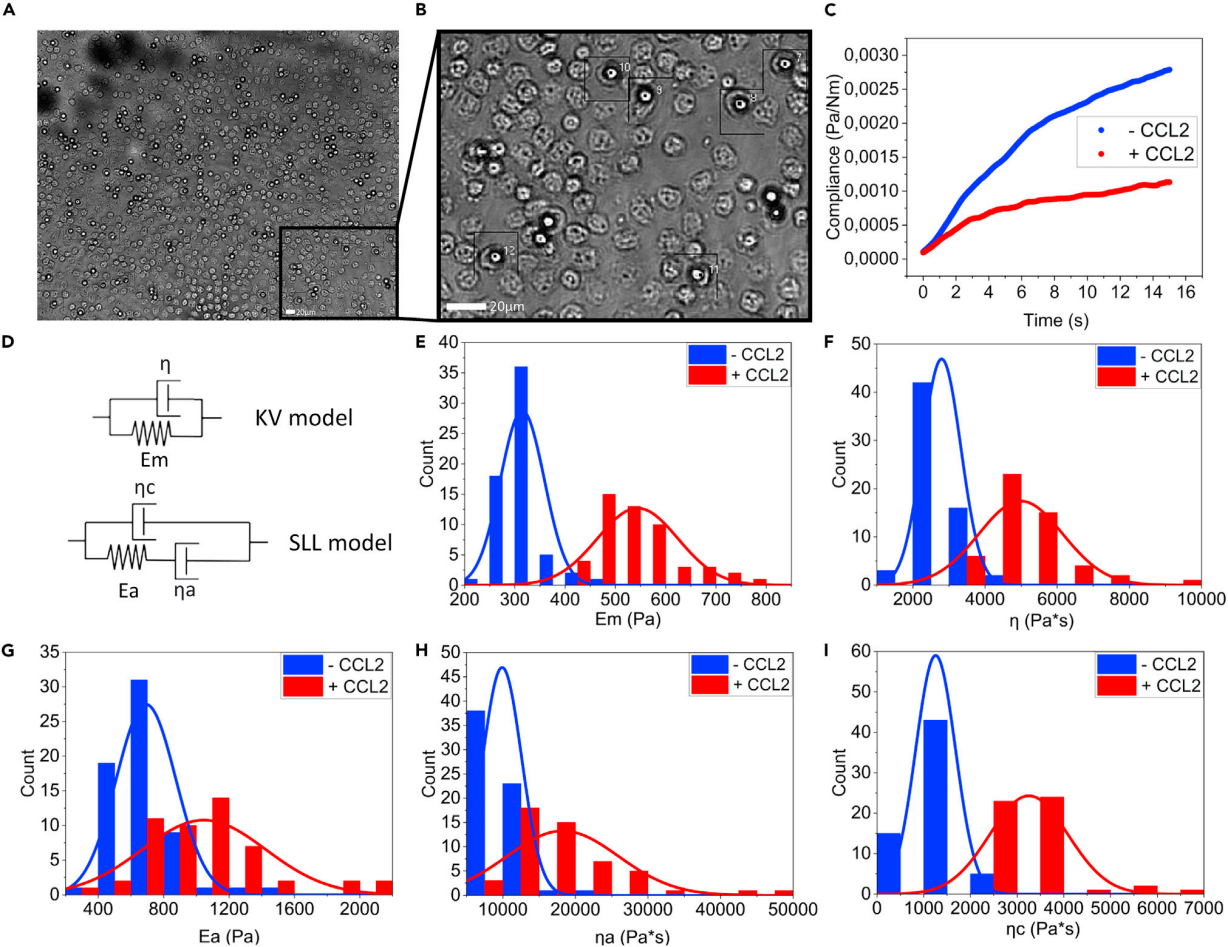
CCl2 alters the viscoelastic properties of human monocytes, measured with AFS (A) Magnified image of the flow chamber showing a typical field of view in experiments. Tens of beads on top of the monocytes are individually tracked. (B) Further magnified image of the typical field of view showing single monocytes with beads attached on top. (C) Typical trace (creep compliance) of a monocyte in response to the application of a constant force (3.5 nN) at physiological temperature and after exposure to CCL2. (D) Schematic representations of the KV and SLL model. (E) CCL2 induces a significant increase in the average elastic modulus E_m_ of monocytes (p < 0.0001). (F) CCL2 induces a significant increase in the viscosity η of monocytes (p < 0.0001). (G) CCL2 significantly increases the average elastic modules Ea associated with the cytoskeleton (p < 0.0001). (H) CCL2 significantly increases the average viscosity ηa associated with the cytoskeleton (p <0.0001). (I) The average background viscosity ηc of monocytes significantly increases upon stimulation by CCL2 (p <0.0001). Distributions plotted using Origin-Lab (2019b). p-values calculated using Mann-Whitney test.

Human monocytes are known to exhibit morphological heterogeneity, such as variability in size, granularity, and nuclear morphology (Gordon and Taylor, 2005). This raises the question whether these cells also show mechanical heterogeneity, and whether this heterogeneity is also regulated by CCL2. In order to characterize the mechanical heterogeneity of monocytes, we utilized AFS, a method that complements OT in experimental throughput. In our experimental setup, freshly isolated human monocytes are confined between concanavalin-A functionalized silica beads (7.9 μm in diameter) and the glass surface of the AFS microfluidic chip (Figures 2A and 2B). Acoustic forces are applied via a piezo element, which generates standing acoustic waves that push the beads up toward the acoustic node, instantaneously stretching the cells at an approximately constant stress $\sigma_{0}$; the OT experiment is therefore considered to expose the cells to a step stress. Bead movement is tracked in real time, where the z-directional displacement corresponds to the elongation of the cell, a quantity directly related to the strain. Only beads that are attached on top of cells are included in the analysis, as beads that are attached to the side of a cell might interact with the surface which can affect the results.

In response to acoustic forces, human monocytes show a typical creep response: an initial regime of sublinear viscoelastic response followed by the slow linear increase characteristic of purely viscous response. From our stress-strain curves, we first calculated the creep compliance $J\left(t\right)=\gamma \left(t\right)/\sigma_{0}$, i.e., the total load strain per unit of stress for each monocyte over a time span of 60 s. From the creep compliance (Figure 2C), we determined the viscoelastic properties. We employed specific viscoelastic models to describe the viscoelastic behavior of monocytes. The best-fitting viscoelastic model for the full time range of 60 s was the Kelvin-Voigt (KV) model (Figure S3), which places one spring, with elastic modulus E_m_, in parallel with a dashpot, with viscosity η (Figure 2D). After stimulation with CCL2, we observed marked alterations in the mechanical phenotype of monocytes. We found significant increases in the average elastic modulus E_m_ and viscosity η, suggesting —as our OT experiments did —that CCL2 alters both the elastic and viscous moduli of monocytes (Figures 2E and 2F).

Because the KV model places particular emphasis on the later-time regime of viscous deformation, and we are particularly interested in the short-time mechanical changes monocytes experience when exposed to CCL2, we now zoom in on the short-time regime (t <15s) using the more elaborate standard linear liquid (SLL) model for viscoelastic response. This SLL is comprised of a spring Ea and a dashpot ηa arranged in parallel with a background viscous medium with viscosity ηc (Figure 2D). The reason for choosing this model is that it allows, in principle, to distinguish the contributions to the overall viscoelastic response from the cytoskeleton (Ea and ηa) and the cytosol (ηc). The KV model groups the viscous contributions from cytosol and cytoskeleton into one effective viscosity. In light of the purported mechanism of action of CCL2, we attempt to disentangle these contributions. At the level of springs-and-dashpots models, this requires modeling the cytoskeleton as a viscoelastic liquid (Maxwell element) and the cytosol as a (Newtonian) viscous liquid, deforming in parallel with it (Rodriguez et al., 2013). As such, the SLL mode is the simplest viscoelastic liquid model with the two contributions we are interested in here. Other viscoelastic models, notably the standard linear solid (SLS) model, are also frequently used to model cell mechanics. We have tried to fit our compliance measurements with the SLS as well, and while this yielded similar goodness of fit as the SLL, it features an initial, instantaneous elastic response manifested by a jump in the compliance at t = 0, and is therefore unable to reproduce the fact that our compliance curves all start at, or very close, to zero. It appears therefore that our monocytes, like— for instance— the macrophages, studied in (Ekpenyong et al., 2012) are systematically better fit by the SLL (Figure S3). Other viscoelastic models, such as the four-parameter Burgers model, were discarded because of the potentially confounding effect of a larger number of parameters which, moreover, do not permit us to disentangle the mechanical contributions from the cytoskeleton and the cytosol as straightforwardly as the SLL. We also note that even for the SLL, this distinction is not perfect; contributions from the membrane and the overall incompressibility of the cell on these short timescales are folded into the three SLL parameters; it will nonetheless prove instructive to view the short-time data through this lens.

Fitting the SLL to the short-time data, we observed large increases in the average elastic modulus Ea, and in the mean viscosity ηa (Figures 2G and 2H). At the same time, we observed significant changes in the background viscosity ηc (Figure 2I), strongly suggesting that CCL2 acts on the cytoskeletal network, as well as the viscoelastic properties of the cytosol. Moreover, we observe that the distributions of Ea and ηa (Figures 2E and 2F) significantly broaden upon exposition to CCL2 (p < 0.0001 and p < 0.0001 respectively), suggesting that in addition to their averages, the heterogeneity of the mechanical moduli and viscosities, quantified by the range of observed values, is responsive to CCL2. This broadening effect shows high reproducibility between different experiments (Figure S5).

The actin network organization largely determines the global mechanical properties of the cell, including cell morphology and stiffness. In this study, we observed that monocytes treated with CCL2 undergo morphological alterations within a time span of 15 min (Figure 1B), suggesting that they rapidly reorganize their cytoskeleton. Indeed, monocytes treated with CCL2 colocalize actin and tubulin filaments in filopodia, projecting in the direction of their migration (Carvallo et al., 2015). It has been shown that upon stimulation by chemokines, actin filament nucleation occurs allowing *de novo* generation, as well as elongation of pre-existing actin filaments (Hons et al., 2018; Thelen and Stein, 2008). While having not directly quantified the changes in actin density here, we speculate that the CCL2-induced mechanical changes, and the changes to the cytoskeleton may share a common cause: Cytokine-induced upregulation of both the generation and the elongation of actin fibers, absent strong changes to the depolymerization rate, would increase the overall density of the actin cytoskeleton providing a possible mechanical pathway to the observed increases in both the average elastic and viscous moduli. We note, already here, that while the elastic modulus and the viscosity both scale with density the viscosity is determined, in addition, by a molecular-scale relaxation time determined by the dynamics of the crosslinking which may vary independently of density changes; for these reasons the relation between viscosity and density is only valid all else being equal.

We further performed correlation analysis on the mechanical parameters obtained from the SLL model fits applied to AFS data. Spearman-rank correlation matrices for monocytes at PT untreated and treated with CCL2 are demonstrated in Figure S6. Future studies may shed light on the potential role of density and dynamic effects in the cytoskeleton (particularly, crosslinker on/off kinetics) on the correlation between the viscous and elastic properties of human monocytes.

## Discussion

Our findings present the first single-cell force spectroscopic characterization of human monocyte mechanics and unravel a role for CCL2 in modulating the viscoelastic behavior of monocytes. Our observations indicate that (i) monocyte mechanics is strain-rate dependent, (ii) CCL2 increases both the viscous and elastic properties of monocytes, and (iii) monocytes exhibit inherent mechanical heterogeneity, which is regulated by CCL2, suggesting that CCL2 modulates monocytes mechanically to transit to a primed state.

Our analysis reveals important differences in the mechanical properties of monocytes at physiological temperature vs. those at room temperature. Relevant data on monocyte mechanics under physiological temperatures is largely lacking, as previous studies probed the viscoelastic properties of human blood monocytes either at room temperature or by utilizing monocytic cell line models which do not fully recapitulate human blood monocyte physiology and functionality. Although these latter studies provided a useful approximation of the mechanical behavior of monocytes, our data emphasize the need to probe cell mechanics in temperature-controlled environments.

**Figure 3:**
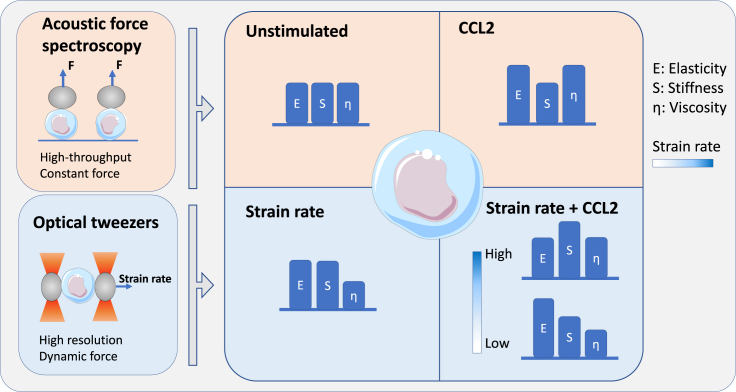
Graphical summary of the effects of strain rate and CCL2 treatment on monocyte mechanics, measured with OT and AFS. The figure illustrates how two fundamentally distinct modes of regulation, namely regulations through chemical and mechanical stimulations modulate monocyte mechanics. Mechanical strain increases the stiffness and elastic modulus, and decreases the viscosity of monocytes. Chemical stimulation by CCL2 induces an increase in both elastic modulus and viscosity. Simultaneous mechanical and chemical stimulation of monocytes lead to increased elastic modulus at lower stretching velocities, and increased viscosity and stiffness at higher velocities.

Our data indicate that mechanical strain and chemical stimulation by CCL2 work synergistically to regulate the mechanical properties of monocytes. This regulation is buttressed by the fact that modulation of viscous and elastic properties are related to time scales underpinning dynamic control, leading to physiological resting versus primed states (Lautenschläger et al., 2009). For this regulation, we propose a model, illustrated in Figure 3. Circulating monocytes have to repeatedly squeeze through microvasculature constrictions smaller than their diameter while being advected along at high velocities. Surprisingly, chemical stimulation of monocytes by CCL2 increases both elastic modulus and viscosity and broadens respective distributions, suggesting that CCL2 primes monocyte mechanically, thereby potentially influencing the swiftness of the response to pathogenic immune triggers.

Because we used different cell populations for the CCL2- and CCL2+ assays, we cannot comment on the absolute changes induced by CCL2 that might cause the broadening of the distributions. The action of CCL2 is likely to multiply the elastic modulus and viscosity by some factor, which would indeed shift their distributions to both higher means and larger variances (broader distributions). These very finely tuned combinatorial mechanical properties may serve as physical biomarkers for immune activation.

### Limitations of the study

We used simple mathematical models to extract biological information from our OT- and AFS-based single-cell data. Despite the SLL model allows, in principle, to distinguish the contributions to the overall viscoelastic response from the cytoskeleton (Ea and ηa) and the cytosol (ηc), it cannot capture the full complexity of the system. In addition, we linked our CCL2 findings to changes in the actin cytoskeleton, which agrees with previous fluorescence studies, but there might be other contributions as well through other cellular elements.

## STAR★Methods

### Key resources table

**Table:** 

Reagent or Resource	Source	Identifier
**Critical Commercial Assays**		

CD14MicroBeads, human for 1×10^9^ total cells	MiltenyiBiotec	Cat# 130-050-201
AutoMACSpro	MiltenyiBiotec	Cat# 130-092-545
AutoMACS columns	MiltenyiBiotec	Cat# 130-021-101
autoMACSRunning Buffer – MACS Separation Buffer	MiltenyiBiotec	Cat# 130-091-221
autoMACS Washing Solution	MiltenyiBiotec	Cat# 130-092-987
MACS SmartStrainers (30 μm)	MiltenyiBiotec	Cat# 130-098-458

**Other**		
RPMI 1640 Medium, GlutaMAXSupplement (No Hepes)	ThermoFisher Scientific	Cat# 61870036
Penicillin Streptomycin	ThermoFisher Scientific	Cat# 15140122
FetalBovine Serum	ThermoFisher Scientific	Cat# 26140079
Ficoll-Paque PLUS	Sigma-Aldrich	Cat# GE17-1440-02
Greiner LeucoSep-tubes	Sigma-Aldrich	Cat# Z642843
ConcanavalinA from *Canavaliaensiformis*	Sigma-Aldrich	Cat# C5275
Non-functionalized silica microspheres 4.0μm	Bangs Laboratories, Inc.	Cat# SS05002
Silica Particles, 5.0% w/v,6.0-8.0 μm	Spherotech, Inc.	Cat# SIP-60-10
Phosphate Buffered Saline 1x	Sigma-Aldrich	Cat# D8537

### Resource availability

#### Lead contact

• Further information and requests for resources and reagents should be directed to and will be fulfilled by the lead contact, Alireza Mashaghi, (a.mashaghi.tabari@lacdr.leidenuniv.nl).

#### Materials availability

• This study did not generate new unique reagents.

### Experimental model and subject details

#### Monocyte isolation and preparation

Primary monocytes were isolated from healthy donor’s buffy coats. The use of buffy coats for research purposes was approved by the Institutional Review Board of the Leiden University Medical Center, the Netherlands. Buffy coats were collected from healthy anonymous blood bank donors (Dutch, adults) that had signed written informed consent for scientific use of blood products. Peripheral blood mononuclear cells (PBMCs) were obtained by density gradient centrifugation of the donor sample using Ficoll. Monocytes were subsequently isolated by positive selection with a monocyte (CD14+) isolation kit using MACS microbeads and columns according to manufacturer’s protocol (MiltenyiBiotec, GmbH). Purity of the CD14+ fraction was assessed with flow cytometric analysis. For AFS measurements, the concentration of the monocyte suspension was adjusted to 25∗10^6^ cells/ml to ensure optimal working coverage of the chip, whereas OT required a significantly lower concentration.

### Method details

#### OT setup and manipulation cycles

We set up a single-cell optical tweezers assay to measure monocyte mechanics. In our experimental setup, the cell of interest was firmly attached to two separate ConA-coated silica beads (∼4μm, silica beads, Bangs-lab) from its diagonal sides, as schematically shown in Figure 1A. In the final experimental assay both silica beads were optically trapped and cells were kept approximately 4 μm away from the surface in order to prevent possible effects of hydrodynamic forces on cell mechanics. Considering the temperature dependency of water viscosity and heterogeneity in size of the beads, the traps were calibrated in room or physiological temperature (depending on experimental condition), before attaching the beads to the cell, for each set of measurements. Notably, the size of the trapped beads was chosen so that the cell was not directly exposed to the laser beam, even in the most displaced position (diameter of focused laser beam at the center of the trap was less than 800 nm).

Force spectroscopy measurement was conducted in a cyclic manner, in which the cell is periodically subjected to an external tensile force followed by a relaxation time. More specifically, the manipulation cycle consisted of four separate steps: Step 1 (stretch): the cell was stretched with a pre-known strain rate. This was done by moving laser trap 1 with a pre-known velocity for 500 nm (constant strain). Step 2 (relaxation): for 15 seconds. In this step, the cell relaxes the exerted tension and reaches mechanical equilibrium. Step 3 (retraction): trap 1 was returned to its initial position by 500 nm at the same velocity used in step 1. Step 4 (relaxation): The trap 1 was kept stationary for over 15 seconds in order to let the monocytes relax, again. During the entire process the force experienced by trap 2 was monitored at a sampling rate of 10kHz. All measurements were conducted using a C-trap (LUMICKS, Amsterdam) optical tweezers machine and data were analyzed using a homemade script in Python.

#### AFS setup and specifications

AFS-G2 from LumicksB.V. comprises a motorized z-stage mounted on an inverted Nikon microscope (Eclipse, TE200), G2AFS chip holders (microfluidic devices), and a temperature controller. Illumination was provided by a red-light LED and video collected with anuEye (UI-324xCP-M, IDS) camera recording at 60 Hz. A 20× microscope objective was used to image and illuminate the beads. Acoustic force was applied via the LabVIEW software provided by LumicksB.V., which allows for real-time, x, y, and z tracking of bead location. The resonance frequency used for all experiments was 14.37 MHz.

#### Stokes force calibration of AFS chip

A critical aspect in AFS measurements is proper calibration. In order to convert generated voltage signals to force values for the 7.9 μm-diameter silica beads, stokes force calibration (SFC) is required. In our experimental set up, several forces act on the beads; the acoustic force, the gravitational force, the buoyancy force, the drag force caused by the viscous friction in medium, and the inertial effects due to acceleration. The sum of all forces acting on the bead is equal to m∗a, where m is the bead mass and a is the bead acceleration. In our experiments, the bead acceleration is several orders of magnitude smaller than the gravitational acceleration. Hence, the inertial effects due to acceleration can be neglected in comparison to the gravitational force. This statement stands not only for the bead but also for any part of the cell.

The acoustic force is the driving force which makes the bead move, but also affects the cell. As theoretically calculated in (Settnes and Bruus, 2012), the bead experiences the acoustic force to a much larger extent than the cell. We also observed this effect experimentally; at some acoustic forces the bead shoots up very fast, while the cell does not show any sign of deformation. Hence, it is safe to say that the cell experiences only the pulling force from the bead.

The acoustic force results from the standing acoustic wave and pushes the bead towards the acoustic node. This force is maximal at the anti-nodes and is zero at the nodes. Since the bead displacement is much smaller than the wavelength of the standing acoustic wave, one can consider the acoustic force as constant. The acoustic wave amplitude is therefore proportional to the voltage applied to the piezo-element which excites the wave, and hence to the square of the applied voltage:

(Equation 1) $$F=\text{ c}V^{2}$$

Where F is the acoustic force, V the voltage applied to the piezo-element and c the conversion factor.

The conversion factor is found by a setup calibration. During calibration, i.e., without a cell, a single bead is subject to gravitational, acoustic, and drag forces according to:

(Equation 2) Facoustic=Fstokesdrag+Fgravity–Fbuoyancy

(Equation 3) $$Facoustic=\left(\mu_{bead}\gamma_{Brenner}\right)+\left(\frac{4}{3}\text{π g }r^{3}\rho_{bead}\right)–\left(\frac{4}{3}\text{π g }r^{3}\rho_{medium}\right)$$

With

(Equation 4) $$\gamma_{Brenner}=\;\frac{6\text{π}\eta r}{1-\frac{9r}{8h}+\frac{r^{3}}{2h^{3}}-\frac{57r^{4}}{100r^{4}}+\frac{r^{5}}{5h^{5}}+\frac{7r^{11}}{200h^{11}}}\;$$

where g is gravity, r is the bead radius, ρ_bead_ is the density of the silica bead, ρ_medium_ is the density of PBS, u_bead_ is the bead velocity, γ_Brenner_ is the correction factor for Stokes drag coefficient, η is the viscosity of the medium, and h is the height of the bead center to the surface. Brenner’s drag coefficient was determined by measuring the viscosity of the bulk fluid and directly inserting it into Equation 4. In order to find the experimental local viscosity, η, and to use it for the determination of the effective drag coefficient, γ_Brenner_ a method based on the terminal velocity of the bead was employed. The method works by pushing the bead towards the acoustic node and then determining the viscosity of the fluid by tracking the terminal velocity of the bead as it settles. The terminal velocity is reached when the velocity more or less remains constant (Figure S1A). The viscosity of the media used here, PBS at 37°C, was determined to be 0.76 ± 0.07 mPa s (Figure S1B).

For determining the conversion factor and the acoustic radiation force, silica beads (Spherotech, Inc., SIP-60-10) were suspended to a concentration of 0.5 mg/ml in PBS and injected in the chip. Temperature was set using the LabVIEW software. For each bead inside the field of view a look-up-table (LUT) between each bead’s radial intensity profile and its z-position was generated. To displace the beads from the chip surface three different acoustic amplitudes (4%, 5% and 6%) were applied for 1 sec (Figure S1C). The time between applying these amplitudes was about 5 sec for the SFC in PBS. Typically, about 4-5 beads at random positions were measured simultaneously inside a field of view. For our measurements, we kept the field of view in the same position and avoided corners. This is essential as Nguyen et al. extensively showed that the force distribution in the chip can vary depending on the position of the bead (Nguyen et al., 2021). The spatial calibration map (n = 20 beads) for our field of view obtained by performing the Stokes force calibration is shown in Figure S2. The average conversion factor c in our field of view was determined to be 0.71 ± 0.095 (Figure S1D).

#### Loading of the AFS flow chamber

Fresh monocytes were injected (0.200 μl/min) into the flow chamber of the AFS chip, which is mounted on the AFS microscope setup. The chip is controlled to physiological temperature. After closing the outlet valve to prevent flow during the cell incubation time, monocytes precipitate and then non-specifically adhere to the bottom glass surface. Following an incubation time of approximately 1 min, the valve is reopened to remove unattached cells, while allowing fresh cells to enter the chip. This process is repeated until a desired number of cells has adhered to surface inside the calibrated field of view. Next, silica beads are introduced into the flow chamber, precipitate and attach on top of monocytes. The outlet valve is closed to allow firm adhesion of the beads to the cells (±5 mins). After generating the LUT for each bead in the field of view, a constant acoustic force regime inside the AFS chip is applied in an increasing fashion, pulling the beads up-wards, thereby stretching the monocytes.

#### Determining the viscoelastic properties of monocytes

The viscoelastic properties were determined by fitting to the standard linear liquid (SLL) model (for the short times, t<15s) and the Kelvin-Voigt (KV) model (for the full time range of 60s). The creep compliance J(t), as a function of time, in the SLL model is given by

(Equation 5) $$J\left(t\right)=\left(\frac{t}{\eta a+\eta c}\right)+\left(\frac{{\left(\eta a\right)}^{2}}{Ea{\left(\eta a+\eta c\right)}^{2}}\right)\text{∗ }\left(1-e^{\left(\frac{\frac{-t}{\text{ ηa∗ηc}}}{Ea\left(\text{ηa}+\text{ηc}\right)}\right)}\;\right)\;,$$

where Ea is the elastic modulus associated with the actin cytoskeleton, η_a_ the viscosity associated with the actin cytoskeleton and η_c_ the background viscosity. In the KV model, the short-time behavior of the same creep compliance is fitted to the simpler expression

(Equation 6) $$J\left(t\right)=\frac{1}{Em}\text{∗}\left(1-e^{\left(\frac{\frac{-t}{\eta }}{Em}\right)}\;\right)\;,$$

where Em is the elastic modulus, and η the viscosity; both now describe the entire cell.

To obtain the data to fit to these expressions, we compute the creep compliance J(t) from our raw data (z-displacement versus time) using

(Equation 7) $$J\left(t\right)=\frac{\text{γ}\left(\text{t}\right)}{\text{σ}}\;,$$

where γ(t) is the strain estimated as the z-displacement over time t divided by is the radius of the bead, and σ the typical stress defined by the applied force (typically 3.5 nN) divided by the area of contact between the bead and the cell (which was in turn calculated by simulating the configuration in our OT setup and imaging the contact from the side, as illustrated in Figure S4). J(t) was then plotted as a function of time and Equations (Equation 3), (Equation 4) and 4 were fit to the resultant curves. The Origin lab fitting tool was used to automatically fit the models to all measured compliance versus time plots.

#### Data acquisition

Data were acquired using the LUMICKSAFS stand-alone technology including a LabVIEW interface for microsphere tracking. All measurements were performed at 37°C, with an amplitude of 70% at 14.37 MHz frequency, applied constantly for a period of 60s. An air 20 ×0.75 NA objective (Nikon, CSI CFI PLAN APOCHROMAT LAMBDA DM20x, PDV,(MRD30205)) was used for tracking bead movement.

#### Silica microbead functionalization

Silica beads (7.9 and 4.0 μm in diameter for AFS and OT respectively, 1% wt/wt;) were first washed by 3% (vol/vol) dilution in PBS, followed by centrifugation at 2000 RPM (for 3 min) and removal of supernatant. Then, beads were surface activated by incubation in 3% HCl (10 min). After washing in PBS and centrifugation at 2000 RPM for 3 min (2x), microbeads were functionalized by incubation with concanavalin A (1 mg/ml; Sigma-Aldrich C5275) for 30 min at 4°C using a tube rotator. Finally, the beads were washed and resuspended in 500 μl PBS.

### Quantification and statistical analysis

Normality of the datasets was tested with a Shapiro–Wilk normality test. For AFS datasets, the non-parametric Mann–Whitney U test was employed to determine significant changes induced by CCL2 exposure. For OT datasets ANOVA was used to determine significant differences between applied strain rates. Data are taken as significant if the p-value is lower than 0.05. For data processing and visualization, Python (version 2.7.15), R (version 3.6.0) and Origin (version 2019b) were utilized.

## Data Availability

• Data reported in this paper will be shared by the lead contact upon reasonable request. • This paper does not report original code. • Any additional information required to reanalyze the data reported in this paper is available from the lead contact upon reasonable request**.**
