# Supplementary material for: Single-cell analysis reveals chemokine-mediated differential regulation of monocyte mechanics
Source: iScience. 2021 Dec 2;25(1):103555. doi: 10.1016/j.isci.2021.103555 (PMC8693412; doi:10.1016/j.isci.2021.103555)
Supplement: Document S1. Figures S1–S6 [file mmc1.pdf]

iScience, Volume *25*

Supplemental information

Single-cell analysis reveals chemokine-mediated differential regulation of monocyte mechanics

Tom M.J. Evers, Vahid Sheikhhassani, Mariëlle C. Haks, Cornelis Storm, Tom H.M. Ottenhoff, and Alireza Mashaghi

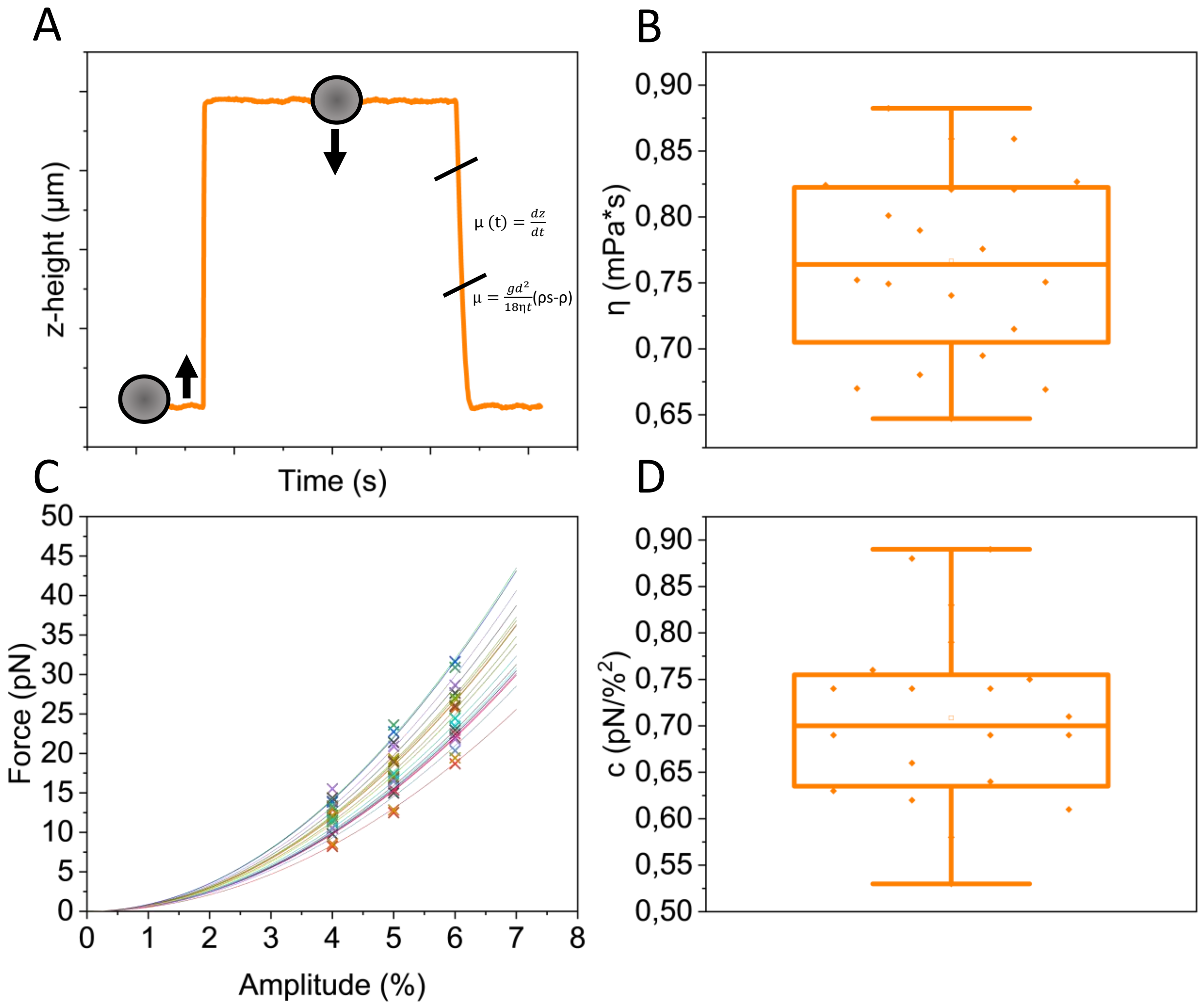

Figure S1. Stokes force calibration of the AFS chip, Related to Figure 2. (A) Determination of the experimental viscosity by tracking the terminal velocity of the bead as it settles towards the bottom of the chip. (B) The viscosity of the media used here, phosphate buffered saline (PBS) at 37 °C, is determined to be 0.76 ± 0.07 mPa*s. (C) Parabolic fits between the acoustic force and the applied amplitude. The force was calculated with the Brenner viscosity correction and the data points (colored crosses) represent the forces for all beads measured (n=20) to obtain the conversion factor c. (D) The average conversion factor for the used field of view is determined to be 0.71 ± 0.095 at a frequency of 14.37 mHz.

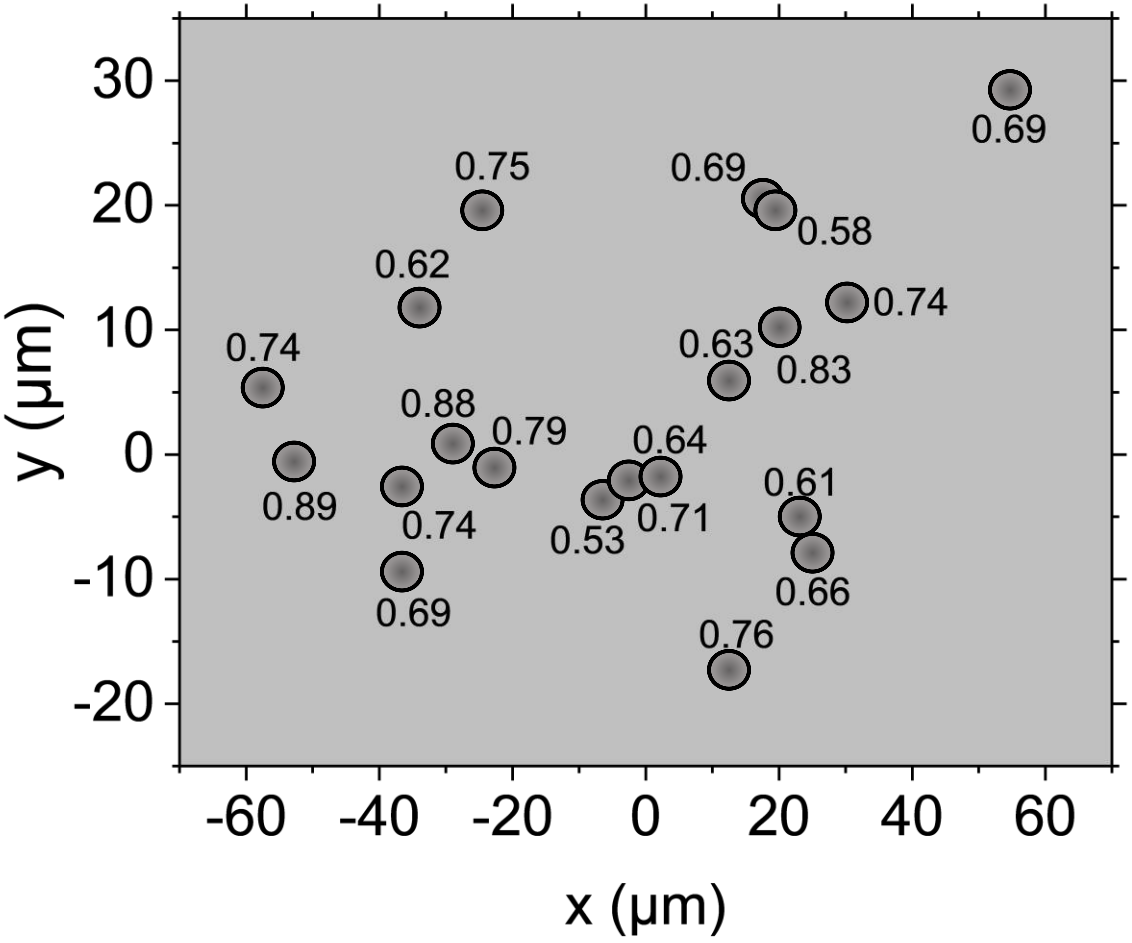

Figure S2. Spatial calibration map (n = 20 beads) for the field of view obtained by performing the Stokes force calibration, Related to Figure 2. The force is equally distributed within the field of view (average conversion factor = 0.71 ± 0.095).

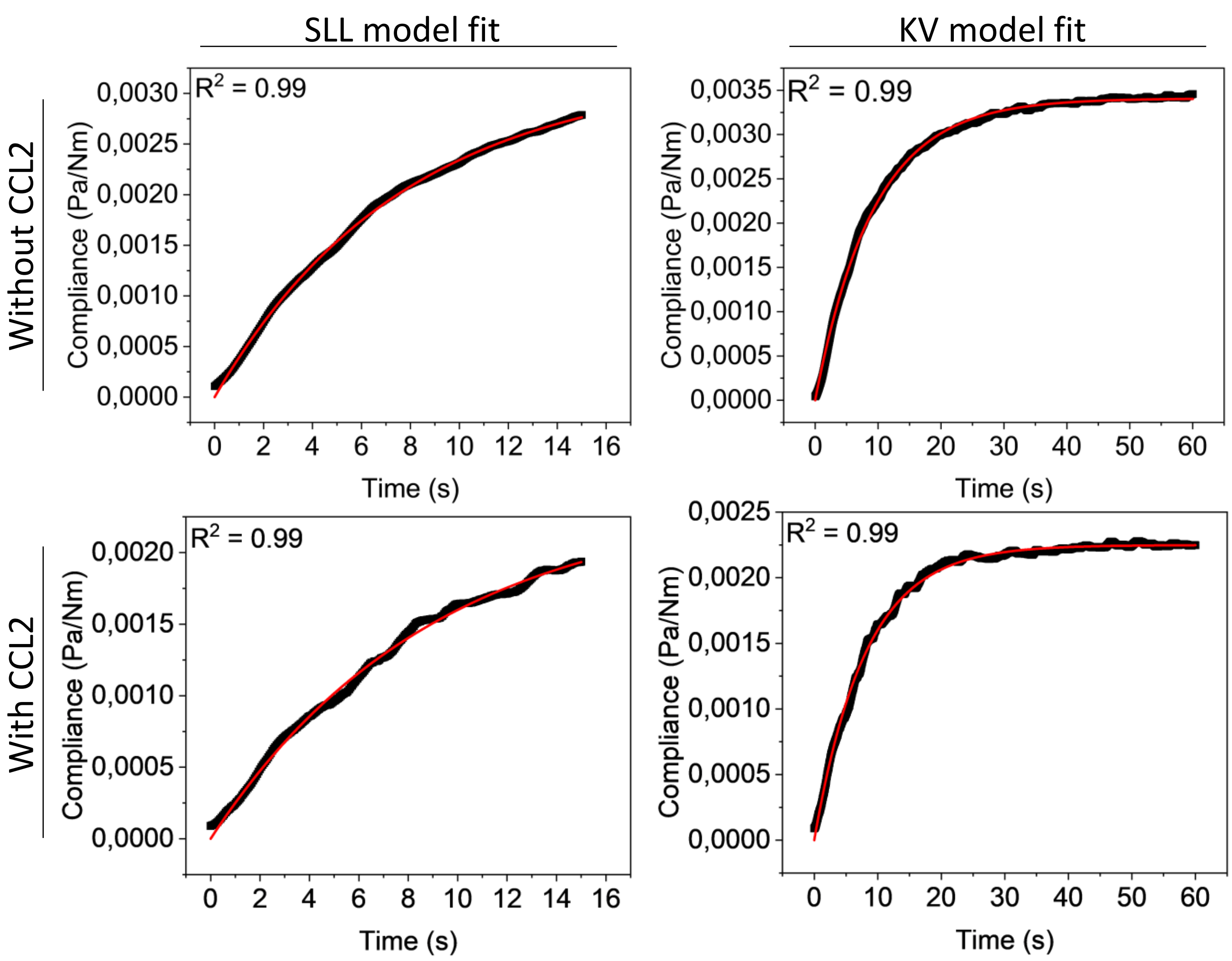

Figure S3. Standard linear liquid (SLL) and Kelvin-Voigt (KV) model fits to creep compliance traces of human monocytes at 15 seconds and 60 seconds, respectively, Related to Figure 2.

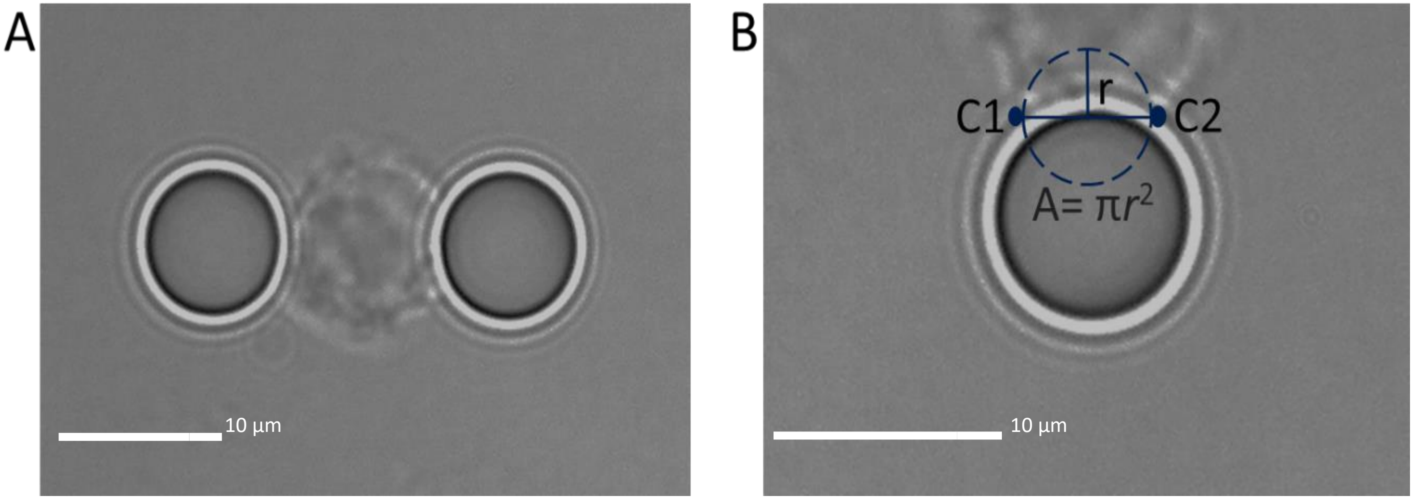

Figure S4. Simulation of the area of contact between a bead and a human monocyte in optical tweezers, Related to Figure 2. (A) Single human monocyte squeezed in between two optically trapped 7.9 µm silica beads. (B) Calculation of the area of contact between contact points C1 and C2 of a 7.9 µm silica bead with a single human monocyte. The average area of contact is determined to be 2.5 ± 0.121 µm (n=5).

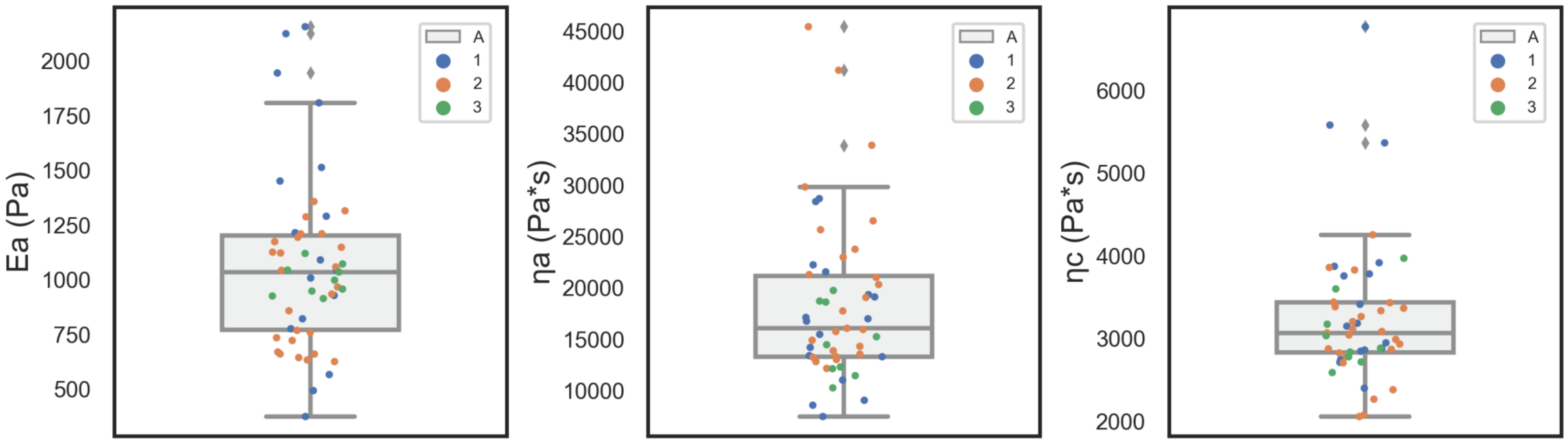

Figure S5. SLL fitted distributions of cytoskeleton associated elastic modulus Ea and viscosity ηa, and cytosol associated viscosity ηc of CCL2 treated human monocytes, Related to Figure 2. Each dot represents a single cell. Each individual repeat of the experiment is shown in a different color (n=3).

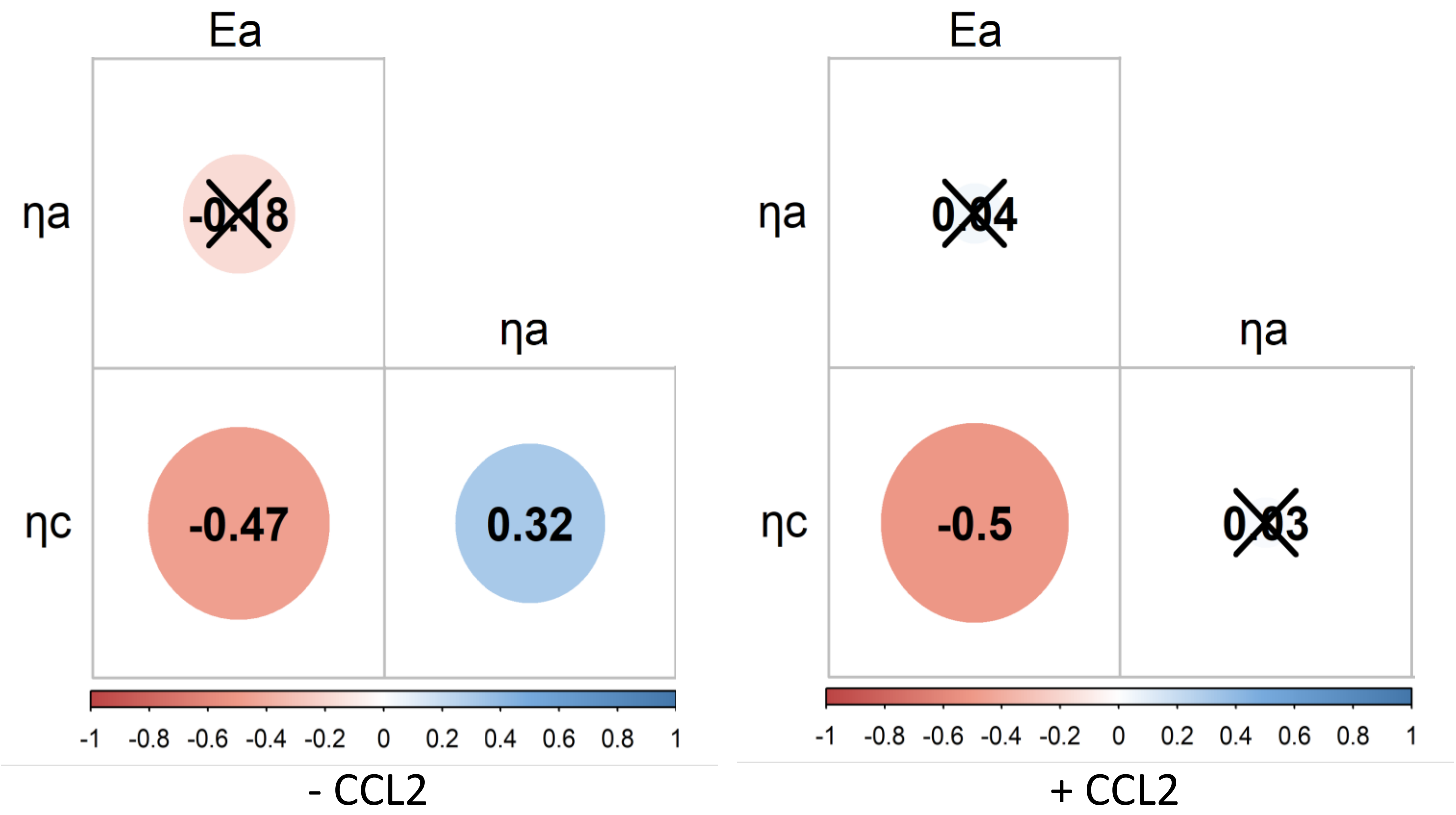

Figure S6. Correlation matrices of monocyte viscoelastic properties (from the SLL model) at physiological temperature and upon stimulation by CCL2, Related to Figure 2. Every correlation coefficient which matches two variables was calculated with Spearman method in R. Numbers ranging from − 1 to 1 are Spearman's rank correlation coefficients of variables on diagonal and vertical axes. Color depth and size of the circles indicates the correlation strengths. Crosses indicate non-significant correlations.
